# Real-Time Prognostics of Engineered Systems under Time Varying External Conditions Based on the COX PHM and VARX Hybrid Approach

**DOI:** 10.3390/s21051712

**Published:** 2021-03-02

**Authors:** Hongmin Zhu

**Affiliations:** Department of Civil and Environmental Engineering, Imperial College London, London SW7 2AZ, UK; h.zhu19@imperial.ac.uk

**Keywords:** prognostics, time-varying covariates, Cox proportional hazards model (PHM), Vector Autoregressive model with exogenous variables (VARX), Conditional Granger Causality (CGC), Fourier Grey model (FGM)

## Abstract

In spite of the development of the Prognostics and Health Management (PHM) during past decades, the reliability prognostics of engineered systems under time-varying external conditions still remains a challenge in such a field. When considering the challenge mentioned above, a hybrid method for predicting the reliability index and the Remaining Useful Life (RUL) of engineered systems under time-varying external conditions is proposed in this paper. The proposed method is competent in reflecting the influence of time-varying external conditions on the degradation behaviour of engineered systems. Based on a subset of the Commercial Modular Aero-Propulsion System Simulation (C-MAPSS) dataset as case studies, the Cox Proportional Hazards Model (Cox PHM) with time-varying covariates is utilised to generate the reliability indices of individual turbofan units. Afterwards, a Vector Autoregressive model with Exogenous variables (VARX) combined with pairwise Conditional Granger Causality (CGC) tests for sensor selections is defined to model the time-varying influence of sensor signals on the reliability indices of different units that have been previously generated by the Cox PHM with time-varying covariates. During the reliability prediction, the Fourier Grey Model (FGM) is employed with the time series models for long-term forecasting of the external conditions. The results show that the method that is proposed in this paper is competent for the RUL prediction as compared with baseline approaches.

## 1. Introduction

Reliability depicts the probability of a system, subsystem, or component to perform required functions within a certain time period under real-life operational and environmental conditions [1,2]. For real-life engineered systems, e.g., manufacturing systems, civil infrastructure systems, and chemical systems, unanticipated failures occurring in such systems will inevitably cause the abrupt breakdown of the systems or their subsystems, which will further give rise to immense costs in maintenance. Thus, it is of vital significance to predict the reliability of the systems under real-life conditions and arrange for future maintenance schedules when considering its advantages of being cost-saving and highly efficient for engineered systems.

As an essential framework in the field of reliability engineering to enhance health monitoring and quality control of engineered systems, Prognostics and Health Management (PHM) has been persistently improved and widely employed in various fields, including mechanical engineering, civil engineering, and chemical engineering, during the past decades. According to [3], the prognostics of failures in engineered systems is proposed to summarise the process of reliability prediction and it occupies an indispensable part of the PHM framework. The aim of the prognostics is to predict the reliability of an engineered system and then infer the failure time of the system based on degradation tests or statistical modelling with access to the failure data [4]. Furthermore, the concept called “Remaining Useful Life (RUL)”, which is assumed as the subtraction between the End of Life (EOL) and the current service time of the system [5], is proposed as the key parameter in prognostics. Within the past decades, many methods for prognostics have been proposed with particular emphasis on the prediction of the RUL.

### 1.1. Failure Prognostics

In terms of the methodology for predicting the RUL, such methods can generally be mainly categorised into three branches [6], namely physics-based prognostics, data-driven prognostics, and the fusion method, which is the combination of the previous two methods.

Among those methods, the physics-based prognostics or Physics of Failure (PoF) based prognostics is regarded as a precise method for investigating the failure mechanism of a system and to then predict the RUL based on prior knowledge of the mechanism [7]. The main advantage of the PoF-based prognostics is its ability to reflect the failure mechanism in specific scenarios and to detect failures in an engineered system [6,8,9]. However, this method exhibits several limitations. Firstly, the method requires parametric information about the properties of the materials or components employed in the system without considering the difficulties that are involved in gaining complete access to the information [6] that may be due to both the real-life operating environment and, sometimes, the confidentiality [8] of data sources. Secondly, the situation to which the physics-based model is applied is restricted to a specific single scenario [6]. Moreover, the method has rigorous requirements for the quality of the data acquired by the degradation tests, thus causing immense costs when conducting the tests [6,10,11]. In addition, under some circumstances, physics-based models, which are established and then further calibrated by means of degradation tests, are not flexible enough to be popularised in the field of prognostics due to their complexity [8,12].

When considering those limitations of the physics-based method, an alternative method, namely data-driven prognostics, is proposed. The essential idea of this method is to predict the reliability or the RUL of an engineered system solely based on the analysis of the failure data without taking the failure mechanism of the system into consideration [13]. To predict the RUL, data-driven prognostics can further be classified into statistical learning prognostics and AI-based prognostics [14], where the former employs statistical approaches, including conventional time series methods, e.g., Autoregressive Integrated Moving Average model, namely the ARIMA model [15,16]; Markov models, particularly the Hidden Markov model [17,18]; the Wiener Process [19,20]; the Proportional Hazards Model [21,22,23,24]; and, other statistical models [25,26,27,28,29]. The latter utilises machine learning methods, such as Artificial Neural Networks (ANNs) [30,31,32,33]; the Support Vector Machine (SVM) [24,34,35,36]; and, others [37,38].

When compared to the physics-based prognostics, the strengths of the data-driven prognostics are embodied within its ability to extract and analyse patterns of the data acquired in the complex engineered systems [39], its high accuracy for RUL prediction [17], and its flexibility for the analysis of various kinds of engineered systems with different characteristics, including multiple variates and nonlinearity [40]. Such strengths enable data-driven prognostics to be a competitive and powerful tool for both condition-based maintenance and predictive maintenance [41].

In spite of the contributions made by the studies that are discussed above, there are several unsolved challenges in the field of failure prognostics with particular emphasis on the accurate RUL prediction of engineered systems. In terms of the external conditions under which the engineered systems perform their functions, the identification of the real-time influence of external conditions (predominantly the Environmental and Operational Conditions, EOCs) upon the reliability functions and the RULs of such systems, including their individual sub-systems and components, has not been conclusively addressed in previous research.

Furthermore, there may exist interdependencies particularly mutual causal relationships from a statistical perspective, between monitoring signals that were measured by sensors arranged in different sub-systems or components. These may, to some extent, reflect how failures of the engineered systems propagate inside these systems. It follows that such information related to the potential failure mechanism can be valuable for an efficient and effective model to be established with high accuracy in so far as it identifies the critical factors having an impact upon the reliability prediction. However, there has been little research in recent years in which a preliminary investigation of the interdependencies between different parts of the engineered systems based upon monitoring sensor signals can define information regarding the potential failure mechanism that would usefully be integrated into the reliability modelling of the engineered systems.

### 1.2. Applications of Vector Autoregressive (VAR) Models to the Failure Prognostics

When considering the potential of Vector Autoregressive (VAR) models as modelling and forecasting tools for multivariate time series, such models have been applied to reliability prognostics in recent years. Li et al. [42], Zhao and Gao [43], and Han et al. [44] similarly employed Principal Component Analysis (PCA) and the VAR for feature extraction and prognostics of faults, respectively. Furthermore, Hochstein et al. [45] proposed a generic framework based on a Regime Switching Bayesian VAR model for prognostics, while Zheng et al. [46] combined the VAR and Granger causality (GC) tests together, a technique that had previously been commonly utilised in econometrics. With this combination, they developed a method to detect and predict computer failures. Moreover, Zheng et al. [46] constructed a topological causal network to make a preliminary investigation of the failure mechanism of computer software.

However, in spite of the potential that the VAR has to integrate external conditions (e.g., ambient temperature, humidity, and operational conditions) into reliability modelling to predict the reliability function and the RUL of the engineered systems, such conditions are not considered in most published publications that are related to the VAR applied to reliability engineering. Although environmental conditions were considered in [42], they were only considered to be endogenous variables rather than exogenous variables, thus potentially causing a misspecification of the statistical model that was built in the research. Actually, the environmental or operational conditions considered to be exogenous factors usually have a negligible influence on the engineered systems, including civil infrastructure systems [47], the aforementioned manufacturing systems [8], and computer systems [48].

Furthermore, most of the research reported in those papers, except Zheng et al. [46], only employed the VAR model as a multivariate tool for diagnostics or prognostics in an engineered system, without further investigating the relationships between different subsystems or components and potential cause–effect failure mechanisms of the system, which can, to some extent, be reflected in the statistical causal relationships (e.g., by means of the GC test) between sensors arranged in different subsystems or components.

In addition, most research in data-driven prognostics, including the applications of the VAR, requires large data sets, which poses challenges to reliability analysis and modelling, based as it is on the available failure data that typically have small sample sizes. In the industry, fierce competition [8] between companies may lead to restricted access to failure data that are considered as confidential information. Furthermore, despite the fact that preventive maintenance enables the early-time replacement of the subsystems or components predicted with failures, it meanwhile hinders the complete “run-to-failure” monitoring of the system’s performance [40], thus influencing the accuracy of the prognostics. In this regard, an effective method for processing limited failure data, including insufficient information of the EOCs, is required. In this paper, the Fourier Grey model (FGM) is utilised to predict the future values of condition monitoring data for the RUL prediction that is based on incomplete failure records.

### 1.3. Survival Analysis Combined with Sensor Signal Forecasting Techniques

Recently the survival analysis technique has been combined with sensor signal forecasting techniques, which, to a large extent, reflect the real-time performance of the engineered systems to yield a real-time estimate of the reliability functions of the engineered systems.In such field, Refs. [35,36] employed the Kaplan–Meier estimation and SVM for prediction of the RUL and the reliability function of engineered systems while Tran et al. [24] employed the Cox proportional hazards model (Cox PHM) with time dependent covariates and the SVM for the same purpose. In addition, Du et al. [49] employed the Vector Autoregressive (VAR) model to predict covariates and then utilised the Cox PHM model to predict the conditional reliability frunction and the RUL that is based on prediction of covariates. However, in terms of the online prediction of the RUL and system reliability, the covariates, namely both the sensor signals and the environmental and operational conditions, may have time-lagging effects on the system reliability and RUL prediction, and such time-lagging effects have rarely been considered in the previous researches. In this paper, the time-lagging effects of covariates on the system reliability are modelled based on the VAR family by taking the reliability index as a dependent variable to be predicted.

The remainder of this paper is divided into the following sections. The second section explains the methodology that was employed in this paper to predict individual reliability functions when considering time varying external conditions.The third part illustrates the implementation of the method that was proposed in this paper, including the data processing on a raw data set in the field of mechanical engineering as case studies. Afterwards, the results are presented and further analysed. Conclusions and future directions for this research are presented in the final section.

## 2. Methodology

### 2.1. Research Framework

**Figure 1:**
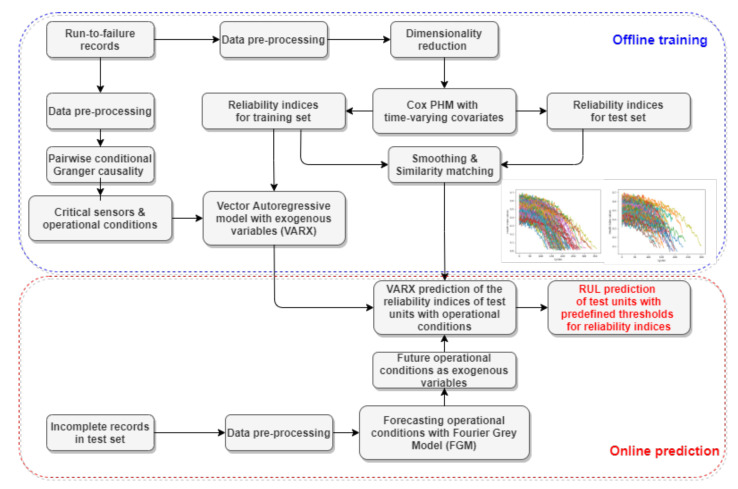
Schematic sketch of the algorithm proposed in this research.

Figure 1 shows a schematic sketch of the algorithm. In the offline training stage, the Cox PHM model considering time-varying covariates is employed to generate reliability indices for different individual failure records after data pre-processing, as shown in this figure. Afterwards, the generated reliability indices are modelled by means of the Vector Autoregressive model with exogenous variables (VARX), together with the critical sensor signals with reduced dimension selected by the pairwise conditional Granger causality test that takes the influence of environmental or operation conditions into consideration. In terms of the online prediction stage, the reliability indices of the incomplete data records are generated by means of the Cox PHM with time varying covariates for further similarity matching. Afterwards, the trained VARX models based on a set of offline failure records, which are most similar to the incomplete records, will be employed to predict future values of reliability indices of the incomplete records and the corresponding RUL with a pre-defined threshold. During the online prediction stage, the Fourier Grey model (FGM) is utilised for predictions of external operational conditions in the incomplete data records that may have small sample size to update the online prediction. The detailed information is described, as follows, in this section.

### 2.2. Cox Proportional Hazards Model with Time-Varying Covariates

Since being proposed in 1972 by Cox [50], the Cox proportional hazards model (Cox PHM) has been regarded as a powerful tool in the field of survival analysis and it has been widely employed in other fields, e.g., medical research [51], economics [52], and reliability engineering due to its competence in modelling the impact of covariates on the survival probability or the reliability of a specific system. In the framework of the standard Cox PHM [50], the hazard function considering a certain set of time-invariant covariates can be expressed in the following formula,

(1) $$h\left(t|x\right)=h_{0}\left(t\right)\exp\left(w^{T}x\right)$$

where h(t|x) represents the hazard function while considering the influence of time-invariant covariates, $h_{0}\left(t\right)$ represents the baseline hazard function that can be estimated by Kaplan–Meier estimation, and *x* and *w* represent the covariates and the coefficients of the covariates, respectively. Correspondingly, the survival function or the so-called relability function while considering time-invariant covariates can be further calculated, as shown in (2),

(2) $$R\left(t|x\right)=\exp\left(-H(t|x)\right)=\exp\left(-\int_{0}^{t}h\left(u|x\right)du\right)=\exp\left(-\int_{0}^{t}h_{0}\left(u\right)du\right)\exp\left(w^{T}x\right)$$

where R(t|x) represents the reliability function considering the influence of covariates, H(t|x) represents the cumulative hazard function, and *t* represents the time during the failure process of an engineered system.

However, in the field of the reliability engineering with particular emphasis on the failure prognostics, the conventional Cox PHM model assuming that its covariates are time-invariant are not intuitively perceivable in the practical sense. Accordingly, the reliability function considering time-dependent covariates is shown in (3),

(3) $$R\left(t|x\right)=\exp\left(-\int_{0}^{t}h\left(u|x\right)du\right)=\exp\left(-\int_{0}^{t}h_{0}\left(u\right)\exp\left(w^{T}x\left(t\right)\right)du\right)$$

where x(t) represents the covariates that are dependent on the time *t* and other items remain the same with those in (2). Furthermore, the time-dependent covariates can be further classified into the time varying covariates and covariates with time varying coefficients [53], where, in the latter case, the covariates themselves are assumed to be fixed, but their influence on the system reliability is time-varying. In this paper, the Cox proportional hazards model (Cox PHM) with time-varying covariates is established to generate the reliability indices of both the run-to-failure records in the training set and the incomplete right-censored failure records in the test set. Please note that it is theoretically difficult to directly generate reliability functions as the reliability indices of both the training set and the test set for comparison when considering the right-censoring of the test set [53]. In this regard, an exponential relative hazards function I(t|x) in the form of $\exp\left(w^{T}x\left(t\right)\right)$, as shown in (4), is proposed in this paper as a reliability index for both the training set and test set. The proposed reliability index is able to reflect the dynamic influence of the time-varying covariates on the system degradation behaviour prior to failure.

(4) $$I\left(t|x\right)=\exp\left(-\exp\left(w^{T}x\left(t\right)\right)\right)$$

### 2.3. Similarity Matching of Generated Reliability Indices

**Figure 2:**
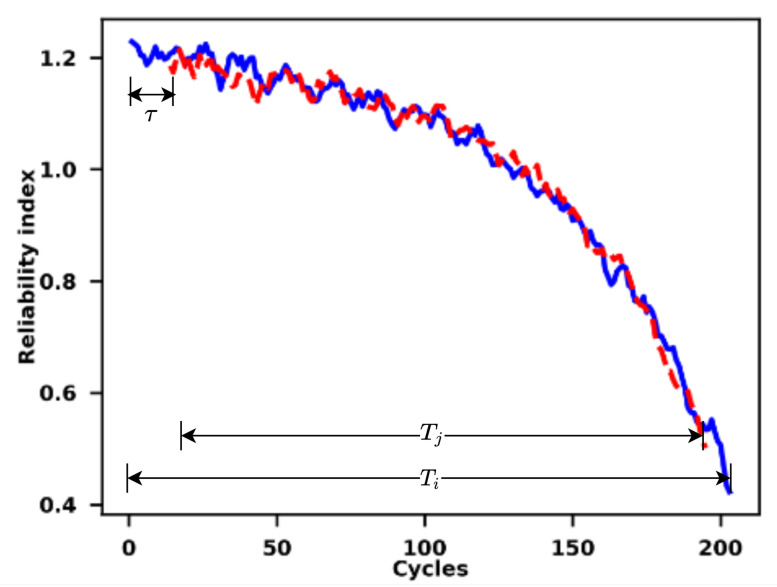
Illustration of the similarity matching process of relaibility indices.

The similarity-based matching between the reliability indices of the training set units and the test set units that are generated by the Cox proportional hazards model with time-varying covariates is utilised in this paper. Figure 2 shows a diagram employed to illustrate the similarity matching process.

The essential idea of the similarity matching is to match the reliability indices of the test units with their corresponding training units that have similar degradation trends based on their distances. According to Wang et al. [54], the distance between two curves of reliability indices is calculated while utilising an Euclidean measure, which is shown in (5),

(5) $$d\left(I^{i},{I^{\prime }}^{j}\right)={\left(\frac{1}{T_{j}}\sum_{m=1}^{T_{j}}{\left({I_{m}}^{i}-{I_{m+\tau }^{\prime }}^{j}\right)}^{2}\right)}^{\frac{1}{2}}$$

where $I^{i}$ and ${I^{\prime }}^{j}$ represent the reliability indices of the training set *i* and the test set *j*, respectively, $T_{j}$ is the length of the real-time reliability indices of the test set *j*, τ represents the time-lag between the reliability indices of the test units and the indices of the training units. Similar to [55], the maximum time lag is set as 30 to prevent mismatching between the reliability indices and save the high computational cost.

Furthermore, the similarity between the reliability indices can be calculated by (6),

(6) $$Similarity\left(I^{i},{I^{\prime }}^{j}\right)=\exp\left(-\frac{d^{2}\left(I^{i},{I^{\prime }}^{j}\right)}{\lambda }\right)$$

where λ is the relaxing parameter, which is set as 0.002 in this paper [56]. For a specific matching between the unit *i* in the training set and the unit *j* in the test set, the RUL of the test unit *j* based on the training set *i* when considering that the time lag τ is calculated by (7),

(7) $${RUL_{j}}^{i}=T_{i}-T_{j}-\tau$$

where the $T_{i}$ is the length of the reliability indices of the run-to-failure training set *i*. Afterwards, the overall RUL of the test unit *j* is calculated by the average of the RULs based on a set of training units that have the largest similarities with the test unit j in terms of their reliability indices, which is shown as (8),

(8) $$RUL_{j}=\frac{1}{N}\sum_{i\in G}{RUL_{j}}^{i}$$

where $RUL_{j}$ is the overall RUL of the test unit *j* and *G* represents the group containing the training units whose reliability indices have the largest similarities with the reliability index of the test unit *j*. *N* is the size of the group *G* and it is set as 5 in this paper to avoid the high computational cost. Meanwhile, while considering the uncertainties when the matching similarities between a test unit with short and right-censored records and a training unit with run-to-failure records, the restriction of the maximum RUL for all the test units is set as 150 [57,58] in this paper.

In this paper, the results that were obtained by the similarity matching will not be directly used for the RUL prediction. The reason for this is that the variations and uncertainties of the future operational conditions for the incomplete data records are ignored in the typical similarity matching algorithm. To address this problem, the similarity matching algorithm is employed here only to generate the reliability indices of both the offline run-to-failure records and the incomplete records for online prediction of the RUL. The results that were obtained by the similarity matching in this paper, mainly the matched failure records and some critical parameters, including $T_{i}$,$T_{j}$, and τ, will be utilised by the VARX model described as follows for futher online prediction of the RUL for the incomplete data records.

### 2.4. Vector Autoregressive Models with Exogenous Variables (VARX)

As a natural extension of the Vector Auto-regressive (VAR) model that enables the integration of the information that is provided by exogenous variables into such models, the VARX model [59,60] is of the following form.

(9) $$Y_{t}=\alpha +\sum_{i=1}^{p}\phi_{i}Y_{t-i}+\sum_{j=1}^{q}\beta_{i}X_{t-j}+\epsilon_{t}$$

As shown in (9), Y represents a *k*-dimensional vector of time series as endogenous variables and X represents an *n*-dimensional vector of time series as exogenous variables. α denotes the constant term of the VARX model. $\epsilon_{t}$ represents the residuals of the VARX model, including independent identically distributed (i.i.d.) vectors that lead to a positive-definite covariance matrix with zero mean. *p* and *q* are denoted as the order of time lags for modelling endogenous variables and exogenous variables respectively, which can be determined by means of the AIC and BIC criteria. In this paper, the Akaike Information Criterion (AIC [61] is deployed to determine the order of the VARX model. $\phi_{i}$ represents the coefficient matrices in front of the endogenous variables with dimension k×k is estimated by means of the ordinary least squares (OLS) technique. Similarly, $\beta_{i}$ represents the coefficient matrices in front of the exogenous variables with dimension k×n. Note that the VARX(p,q) will degrade to the VARX(p,0), namely the Factor VAR (FVAR) model when only the spontaneous influence of the exogenous variables is considered.

More specifically, according to (1) and (9), if the time-lagging effects of covariates, including the operational conditions in this case, are considered, then the hazard function at a time point *t* can be obtained, as follows,

(10) $$h\left(t|x\right)=\alpha^{\prime }+\sum_{i=1}^{p}\gamma_{i}h\left(t-i|x\right)+\sum_{i=1}^{p}{\phi_{i}}^{\prime }{Y^{\prime }}_{t-i}+\sum_{j=1}^{q}{\beta_{i}}^{\prime }X_{t-j}+{\epsilon_{t}}^{\prime }$$

where h(t|x) represents the hazard function at time *t* in (1), which is also an endogenous variable in the VARX model, *p* and *q* represent the order of time lags for modelling endogenous variables and exogenous variables respectively, $Y^{\prime }$ represents the endogenous variables, excluding the h(t|x), namely the sensor signals, X represents the exogenous variables, namely the operational conditions in this paper. In (10), $\gamma_{i}$, ${\phi_{i}}^{\prime }$, and ${\beta_{i}}^{\prime }$ represent the coefficients to be estimated, and ${\epsilon_{t}}^{\prime }$ is the error term to be estimated. Accordingly, if applying (10) to (4), the VARX model is built in (11)

(11) $$I\left(t|x\right)=\alpha^{\prime }+\sum_{i=1}^{p}\gamma_{i}I\left(t-i|x\right)+\sum_{i=1}^{p}{\phi_{i}}^{\prime }{Y^{\prime }}_{t-i}+\sum_{j=1}^{q}{\beta_{i}}^{\prime }X_{t-j}+{\epsilon_{t}}^{\prime }$$

where I(t|x) is the reliability index that is estimated by the Cox PHM with time-varying covariates in (4) and other symbols have the same meaning with (10).

### 2.5. Conditional Granger Causality

The idea of the Granger Causality (GC) test lies in that, if lagged values of one time series denoted as X are valuable to predict current values of another time series Y, the X is then defined as the “Granger cause” [62] of the Y. As an extension of the typical GC test, the idea of the Conditional Granger Causality (CGC) [63,64,65] test is similar to that of the typical GC test, but it considers the influence of third-party mediating variables namely Z when judging statistical causal relationships between pairwise variables. The calculation of the CGC based on the VAR model is described, as follows,

(12) $$\Psi_{X\overset{}{\to }Y|Z}\equiv \ln\frac{\Sigma_{yy,r}}{\Sigma_{yy}}$$

where $\Sigma_{yy}=cov\left(\epsilon_{y,t}\right)$ represents the covariance matrix of the residuals $\epsilon_{y,t}$ of the VAR model, including the variable X as the potential cause of Y conditional on Z, $\Sigma_{yy,r}=cov\left(\epsilon_{y,t,r}\right)$ represents the covariance matrix of the residuals $\epsilon_{y,t,r}$ of the VAR model excluding the variable X.

The CGC test is employed in this research to investigate the interdependencies with particular emphasis on the causal relationships between pairwise sensor signals when considering the remaining sensor signals and external conditions as mediating variables that may have effects on the statistical causal relationships. In this regard, the CGC test can assist with identifying the most critical sensor signals to thoroughly reveal the potential failure mechanism of the engineered systems and further reduce the dimensions of the input variables for the VARX model that is mentioned above. According to (12), the results obtained by the CGC test depend on the lag order of the VAR model. However, the high order conditional Granger causality test requires a corresponding high order VAR/VARX model to be established and it may lead to instability of the estimated VAR/VARX models for the high-dimensional case. When considering that, the causal relationships between sensor signals and external conditions and selection of critical sensor signals as inputs for the VARX model are determined mainly based on a first order CGC test for the sake of simplicity [66].

### 2.6. Grey Model with Fourier Series Calibration (FGM)

Grey Systems Theory was proposed by [67] to analyse complex systems that exist in various kinds of fields, including engineering [18,68,69] and economics [70,71]. The proposed Grey Systems Theory (GST) is mainly composed of two subparts, which are Grey Relational Analysis (GRA) and the Grey Model (GM), respectively [72]. The former is commonly used to infer the unknown relationships between two or more series, while the latter has been widely utilised to predict the behaviour of uncertain and complex systems with limited data sets available, which is closely related to the problem that is addressed in this paper.

Among the GM family, the GM(1,1) model [72] whose formulae are shown as follows, is the most widely employed GM model,

(13) $$s_{k}^{0}+az_{k}^{1}=b\;\left(k=1,2,\cdots ,n\right)$$

(14) $$s_{k}^{1}=\sum_{i=1}^{k}s_{i}^{0}$$

(15) $$z_{k}^{1}=\frac{s_{k-1}^{1}+s_{k}^{1}}{2}$$

where $s_{k}^{0}$ represents the original series with k=1,2,⋯,n (*n* is the length of the whole series), *a*, *b* are the grey parameters to be estimated, and $z_{k}^{1}$ is defined by formula (15).

According to the current literature, the original GM has several disadvantages. The main disadvantage of the GM is its overshooting problems with exceptional data points [68,73] and its instability when processing the series that are mainly dominated by fluctuation or cyclical terms [74]. When considering the disadvantages mentioned above, the Grey Model with the error calibration by the Fourier series (FGM) [75], as a natural extension of the typical GM, is employed in this paper to forecast future values of the external conditions, namely the EOCs, because of its ability to process sequences with nonnegligible fluctuation terms without losing its robustness when forecasting future values of the predicted sequences. Meanwhile, the residuals between the sample values and in-sample predictions by the GM are modified by means of the high-order Fourier series [76]. More specifically, the error calibration by means of the Fourier series is illustrated, as follows,

(16) $$\epsilon^{\left(0\right)}\left(k\right)=\frac{1}{2}a_{0}+\sum_{i=1}^{k}[a_{i}\cos ik\omega +b_{i}\sin ik\omega ]\;k=2,3,4,\cdots ,n$$

(17) $$P=[\begin{array}{cccccc} 1/2 & \cos2\omega & \sin2\omega & \cdots & \cos2k\omega & \sin2k\omega \\ 1/2 & \cos3\omega & \sin3\omega & \cdots & \cos3k\omega & \sin3k\omega \\ 1/2 & \cos4\omega & \sin4\omega & \cdots & \cos4k\omega & \sin4k\omega \\ \vdots & \vdots & \vdots & \vdots & \vdots & \\ 1/2 & \cos n\omega & \sin n\omega & \cdots & \cos nk\omega & \sin nk\omega \end{array}]$$

(18) $$C={\left(P^{T}P\right)}^{-1}P^{T}\epsilon_{0}$$

(19) $$\epsilon_{0}=\left\{\epsilon^{\left(0\right)}\left(2\right),\epsilon^{\left(0\right)}\left(3\right),\cdots ,\epsilon^{\left(0\right)}\left(n\right)\right\}$$

where $k_{0}=\frac{n-3}{2}$ represents the order of the Fourier series, $\omega =\frac{2}{\pi }$ is a parameter that is related to the frequency of the Fourier series, P is the matrix of the Fourier series terms, *C* represents the fitted values of the residuals by means of the Fourier series, and $\epsilon_{0}$ is denoted as the sequence of the residuals at different time points namely $\epsilon^{\left(0\right)}\left(k\right)\left(k=2,3,\cdots ,n\right)$. When considering the limitation of the Fourier series to model random fluctuations of the residuals sequence, the subtraction between the residuals and its fitted sequence by means of the Fourier series is further modelled by means of the Autoregressive integrated moving average (ARIMA) or the Autoregressive moving average (ARMA) model to calibrate the random variations.

## 3. Case Studies

### 3.1. Data Sets Employed in the Research

**Figure 3:**
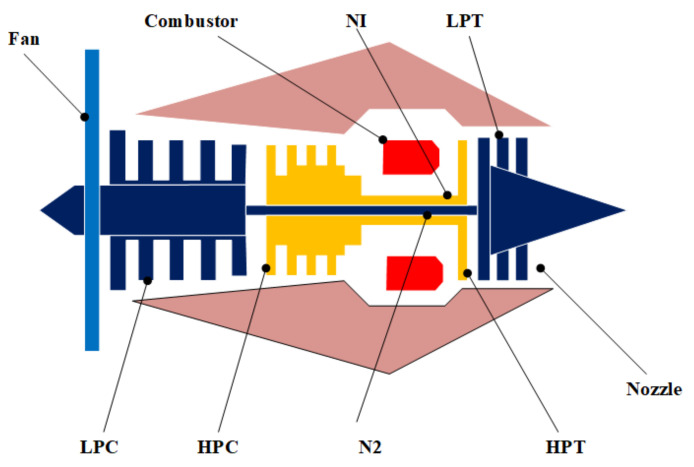
Inner structure of the turbofan employed in the Commercial Modular Aero-Propulsion System Simulation (C-MAPSS) data sets [78].

The NASA turbofan failure data sets simulated by the so-called “Commercial Modular Aero-Propulsion System Simulation (C-MAPSS)” [77] software were employed for case studies in this paper to preliminarily validate the feasibility and effectiveness of the proposed approach. In such data sets, the degradation behavior of the turbofan (as shown in Figure 3) working under three kinds of operational conditions was measured with 21 sensors of different modalities, including temperature, pressure, and speed [77]. The data sets also contain the order of units consisting of the turbofan and time order for data records of each unit [77]. Apart from that, the whole data sets can be separated into four subsets that were simulated under different operational conditions, and each can be further categorised into the run-to-failure training data sets, namely the training sets and the incomplete test sets both with 100 units. In this paper, the proposed approach will be first implemented on the training set of the first subset called the “FD001” data set. After being implemented on the training sets, the model will be implemented on the test sets for prediction of the individual reliability indices and corresponding RULs.

### 3.2. Data Preprocessing

For both the training sets and the test sets, several unchanging columns in them are removed. The remaining data set only contains 2 different operational conditions and 15 different sensor signals. Afterwards, the Min-Max normalisation is implemented on both of the data sets to transfer different scales in all of the time series into a [0,1] scale. A simple moving average filter with a window size of 5 is then utilised to smooth the sensor signals. Because the VARX model and the CGC test assume their input time series to be stationary, the monitoring signals, including the sensor signals and signals of the operational conditions, as well as the generated time series of the individual reliability indices, are again tested for stationarity by means of the Augmented Dickey–Fuller (ADF) tests with the significance level to be 0.05, and all of the non-stationary time series are differenced until they pass the stationary test. In this paper, the order for the difference is set to 1 according to the ADF test.

### 3.3. Implementation of the Hybrid Approach

#### 3.3.1. Implementation of the Cox PHM with Time-Varying Covariates

The monitoring signals in the training set with complete run-to-failure records, including the external conditions, will be utilised by the Cox proportional hazards model (Cox PHM) with time-varying covariates, as mentioned earlier [79], to generate individual reliability indices for RUL prediction based on the failure records of individual units in the training sets. Prior to the reliability modelling based on the Cox PHM model, the Principal Component Analysis (PCA) is implemented on the smoothed monitoring signals to reduce the high-dimensionality of the data. The threshold for the cumulative variance ratio is set as 0.90 and three principal components are selected with their variance ratios to be 0.76, 0.09, and 0.06, respectively. After its training stage based on the training set, the trained Cox PHM model is further implemented on the incomplete test sets to generate incomplete reliability indices of the test set units for further similarity matching.

#### 3.3.2. Implementation of the Pairwise Conditional Granger Causality (CGC) Tests

In terms of the time series modelling of the monitoring signals and the individual reliability functions generated by the Cox PHM approach, the pairwise CGC test [65,80] is then preliminarily implemented to identify several critical monitoring signals, which may greatly influence the reliability indices and, thus, the RULs of the individual units, while considering different external conditions where the units perform their functions.

**Figure 4:**
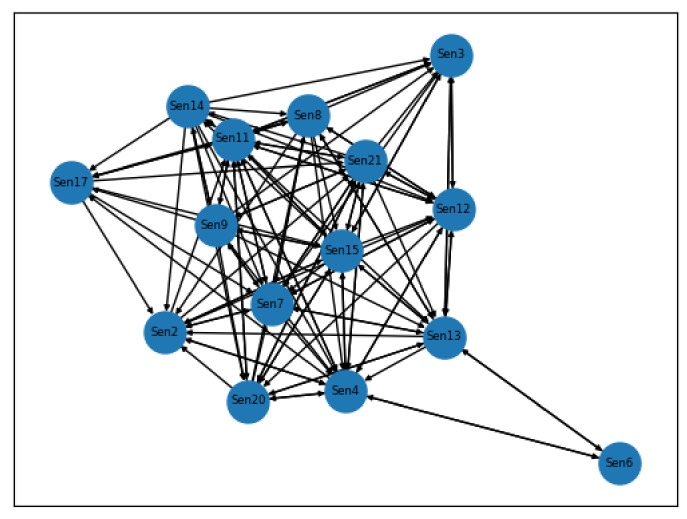
Statistical causal graphs of units in FD001 training set generated by the CGC test.

By means of the CGC tests with the significance level set as 0.05 by this paper, the statistical causal relationships between multivariate sensor signals of the failure data of training set units can be illustrated. For example, Figure 4 shows the first-order statistical causal relationships between different sensor signals in the run-to-failure records of units in the FD001 training sets. The nodes with net outflow greater than 0, namely the 8th, 9th, 14th, 15th, and 21st sensor signals, are defined as critical sensor signals for modelling the degradation behaviour of units in the FD001 training set, which is, to some extent, consistent with the results of the sensor selection mentioned in [81], as shown in Figure 4. For example, the inflow of the sensor signal 9th is 5 while the outflow of this sensor is 10, so the 9th sensor signal whose net outflow is 5 is chosen as one of the critical sensor signals, as shown from the Figure 4. The results obtained by the CGC tests provide a simplified structure with reduced dimensions of variables for the subsequent VARX model to be implemented.

### 3.4. Implementation of the VARX Model

The VARX model with the time series of the external conditions as exogenous variables is then established based upon the previous CGC tests that identify the several critical monitoring signals for further time series modelling of the individual reliability functions and reveal the topological structure of a cluster of sensor signals measured from different parts of the units namely the turbofans. Input variables for the VARX model include the reliability indices generated by the Cox PHM model of individual units and critical sensor signals for degradation modelling of the units as endogenous variables, and the external conditions, namely the operational conditions, where the individual units perform their functions as exogenous variables, as mentioned in Section 2.4.

After estimation of the lag parameters, namely *p* and *q*, a VARX(*p*, *q*) model is employed in this paper to model the relationships between the sensor signals and the time series of the generated reliability indices. When considering that the VARX models for different units in the training set may have different lag orders, different *p* and *q* values are estimated by means of the AIC for differnt VAR models of individual units in training set. In spite of that, the maximum lag order is set as 10 and 2 for *p* and *q*, respectively, to prevent overfitting and instability of the VARX(*p*, *q*) model. The maximum time step for the VARX forecasting is determined preliminarily to be no more than 150 in a similar way to [57,58]. The forecasting process for different units in both the FD001 training sets and test sets will cease once the predicted reliability functions reach the thresholds previously established by the implementation of the Cox PHM model, which generates individual reliability indices of training set units.

### 3.5. Forecast of the External Conditions Using the FGM

Because the time series of the external conditions, namely the environmental and operational conditions (EOCs), as shown in the C-MAPSS data sets, are required to be updated so as to trigger the predictions of the individual reliability indices of test set units by means of the VARX model. WHen considering the large uncertainties and small sample size of the EOCs, the Grey model with error calibration by the Fourier series (FGM) is then implemented to forecast the future values of such external conditions based on the incomplete test sets. The incomplete time series of the EOCs were also normalised into [0,1] scale using the Min-Max normalisation and then smoothed in the same way with the failure data of training set units to avoid non-negative initial values that may lead to instability of the GM.

### 3.6. Prediction of RULs for the Test Set Utilising the Models Based on the Train Set

After reliability indices of the individual units in the training set are generated by means of the Cox PHM model and the generated reliability functions are modelled with the real-time sensor signals of the units in the training set by means of the VARX model with the prediction horizon to be 150 [57,58], the trained model that is based on the training set will be implemented on the test set with the aid of the results obtained by the similarity matching to forecast the RULs of the individual units in the test set. For individual test set units, if their predicted relability indices reach the threshold that is defined as the last reliability index value of their similar training set units, then the VARX prediction is stopped and the corresponding RUL of the test set unit is calculated.

## 4. Results

### 4.1. Cox PHM Fitting Results Based on the Training Set

In this paper, the run-to-failure records of the training set units are fitted by the Cox PHM model with time-varying covariates to generate the reliability indices. Table 1 shows the estimated hazard ratios of input variables and their confidence intervals, *z*-scores, and *p*-values of the input variables of the Cox PHM.

The fitting results show that the first principal component, namely the PC1 in the Table 1, has the largest hazard ratio 3.59 with its *p*-value to be smaller than 0.005, which indicates that the PC1 is a statistically significant factor that has the strongest influence on the degradation behaviour of turbofan units in the training set. When compared to the PC1, the second and the third principal component namely the PC2 and PC3 in this case have weaker influence on the turbofans’ degradation behaviour and are statistically insignificant. Such results are in consistent with the variance ratios of the three principal components, which are mentioned in the Section 3.3.1. According to the variance ratios of the PC1, PC2, and PC3, it can be interpreted that the influence of the PC1 on the turbofan degradation behaviour is much stronger than that of the PC2 and PC3. However, the PC2 and PC3 are stiil contained in the Cox PHM model to hold as much information as possible that is related to the turbofan degradation behaviour based on the complete failure records in the training set.

### 4.2. Results of Similarity Matching of Reliability Indices

**Figure 5:**
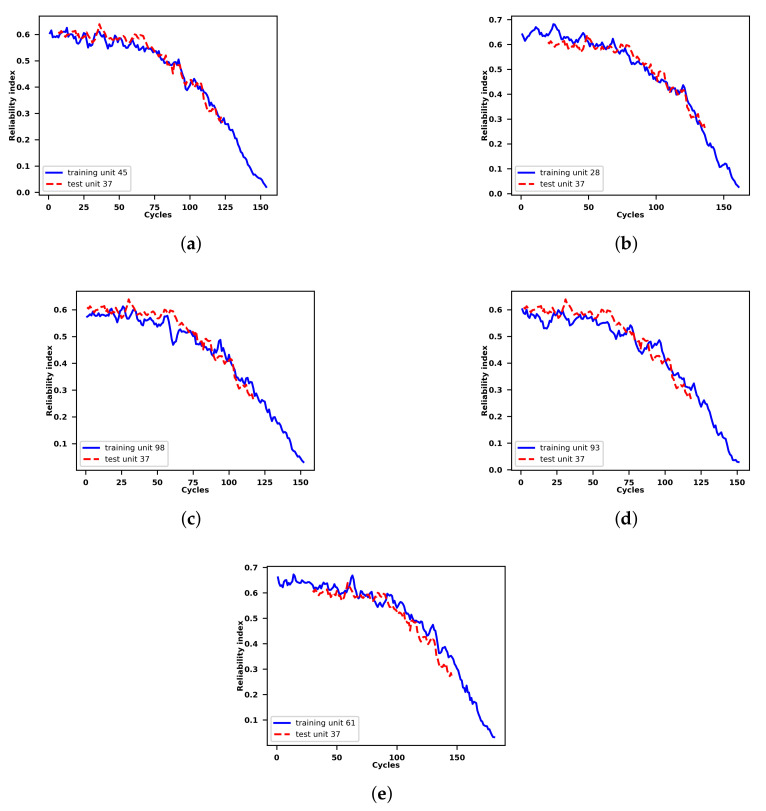
Similarity matching between the incomplete reliability index of the 37th unit in the test set and its five most similar reliability indices of the units in the run-to-failure training set.The similarity decreases with the figure order. (**a**) Incomplete reliability index of the 37th unit in the test set matched with the complete reliability index of the 45th unit in the training set. (**b**) Incomplete reliability index of the 37th unit in the test set matched with the complete reliability index of the 28th unit in the training set. (**c**) Incomplete reliability index of the 37th unit in the test set matched with the complete reliability index of the 98th unit in the training set. (**d**) Incomplete reliability index of the 37th unit in the test set matched with the complete reliability index of the 93rd unit in the training set. (**e**) Incomplete reliability index of the 37th unit in the test set matched with the complete reliability index of the 61st unit in the training set.

As an example, Figure 5 shows the similarity matching results for the incomplete reliability index of the 37th unit in the test set.

According to Figure 5, the relability index of the 37th unit in the test set matches with the 45th, 28th, 98th, 93rd, and 61st unit in the training set from the 7th, 20th, 1st, 2nd, and 30th cycles, respectively. Such information is further employed by the VARX model to predict the future reliability index of a certain unit in the test set based on reliability indices of a set of units in the training set whose reliability indices are most similar to the reliability index of the specific unit in the test set.

### 4.3. VARX Fitting Results

VARX models with different lag parameters for both the endogenous and exogenous variables are established for different training set units, as mentioned in Section 3.4. The overall performance of the VARX models when applied to fitting the in-sample reliability indices based on the failure records of the training set units is plotted in the following figure. In this figure, the Root Mean Square Error (RMSE) and the Normalised Root Mean Square Error (NRMSE) of fitting the generated relaibility indices of training set unit in a single VARX model are both defined in (20) and (21)

(20) $$RMSE_{VARX}={\left(\frac{\sum_{i=1}^{N_{I}}{\left(I_{i}-\hat{I_{i}}\right)}^{2}}{N_{I}}\right)}^{\frac{1}{2}}$$

(21) $$NRMSE_{VARX}=\frac{{\left(\frac{\sum_{i=1}^{N_{I}}{\left(I_{i}-\hat{I_{i}}\right)}^{2}}{N_{I}}\right)}^{\frac{1}{2}}}{I^{max}-I^{min}}$$

where $RMSE_{VARX}$ and $NRMSE_{VARX}$ are the RMSE and the NRMSE of fitting the generated reliability indices by means of a single VARX model, $N_{I}$ represents the length of the generated reliability indices, $I_{i}$ and $\hat{I_{i}}$ represent the actual value and fitted value of the generated reliability indices at the point *i*, $I^{max}$, and $I^{min}$ represent the maximum and minimum value of the actual generated reliability indices.

**Figure 6:**
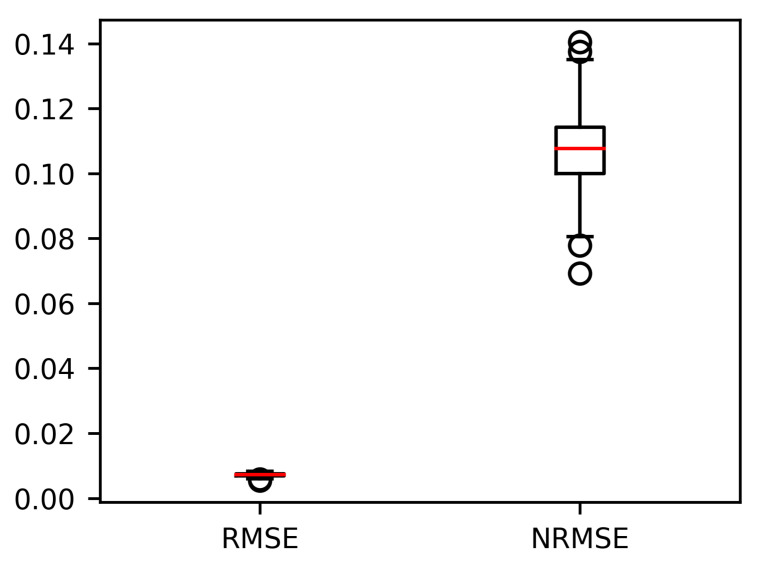
Box plot of the RMSE and the NRMSE of the in-sample fitting of the VARX models based on training set units.

The median value of the RMSEs is 0.0072 with its 25% and 75% percentile to be 0.0069 and 0.0075, respectively, as shown in Figure 6. The median value of the NRMSEs is 0.1077 with its 25% and 75% percentile to be 0.0999 and 0.1142, respectively. The results show that the VARX model has high accuracy when fitting the generated reliability indices of units in the training set.

### 4.4. Fitting and Prediction of the FGM

**Figure 7:**
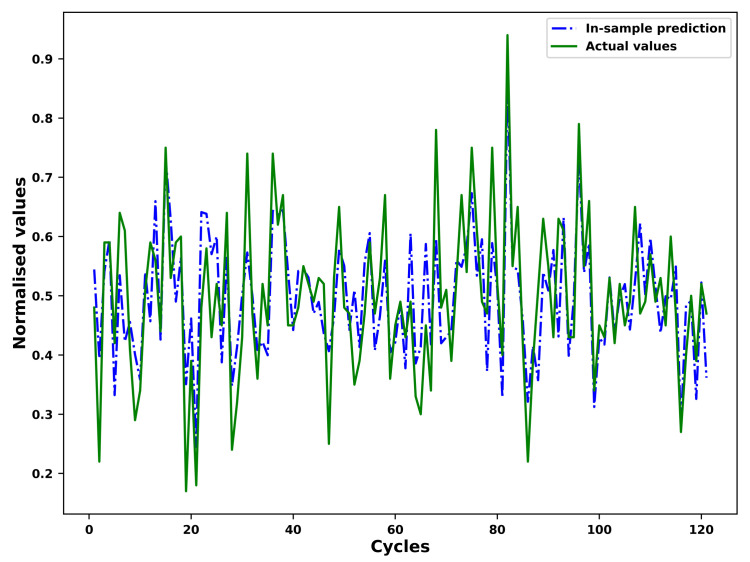
In-sample fitting of the first operational conditions of the unit 37 in FD001 test sets based on the FGM(1, 1) and ARIMA/ARMA calibration.

**Figure 8:**
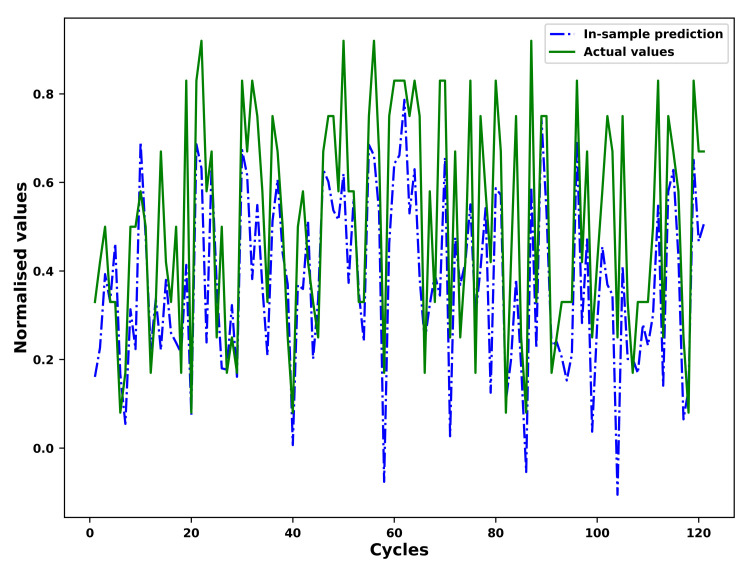
In-sample fitting of the second operational conditions of the unit 37 in FD001 test sets based on the FGM(1, 1) and ARIMA/ARMA calibration.

**Figure 9:**
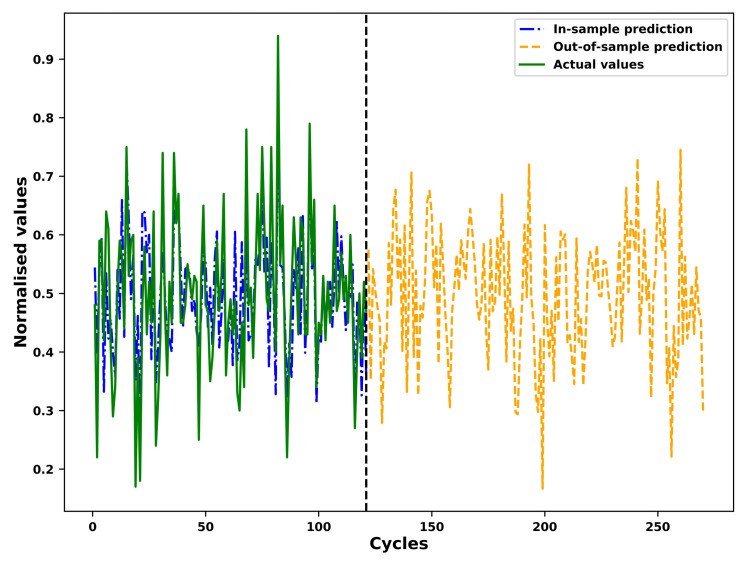
Prediction of the first operational conditions of the unit 37 in FD001 test sets based on the FGM(1, 1) and ARIMA/ARMA calibration.

**Figure 10:**
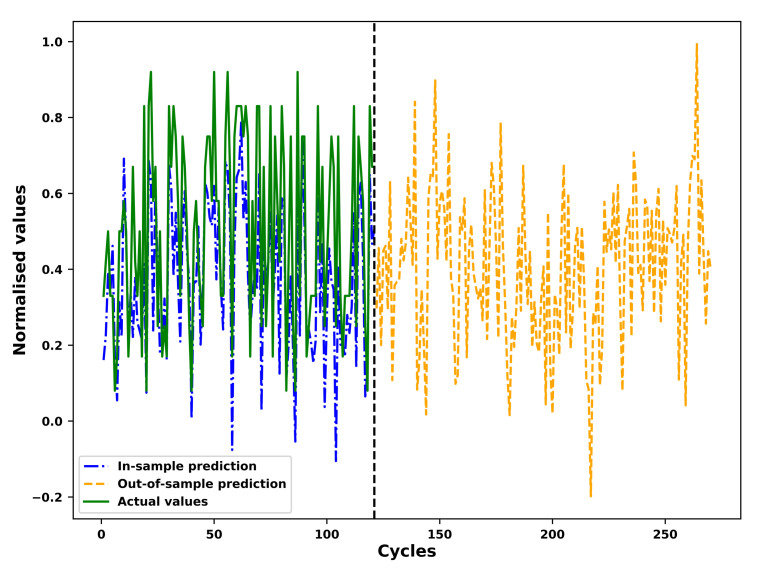
Prediction of the second operational conditions of the unit 37 in FD001 test sets based on the FGM(1, 1) and ARIMA/ARMA calibration.

As an example, the results of the in-sample fitting and the out-of-sample prediction by means of the fourier grey model (FGM) for the different operational conditions of an example unit in the test set is shown in Figure 7, Figure 8, Figure 9 and Figure 10 respectively. As shown in the Figure 7 and Figure 8, the results for in-sample fitting of the first and the second operational conditions for the 37th unit in the test set are illustrated. The RMSE and the NRMSE of fitting the first operational conditions for the 37th test set unit are 0.08 and 0.11 while the RMSE and the NRMSE of fitting the second operational conditions for the same unit are calculated as 0.19 and 0.10, which shows competence of the FGM for in-sample fitting of the actual signals of operational conditions. For all the units in the test set, the mean RMSE and the mean NRMSE of fitting the first operational conditions are calculated as 0.15 and 0.23 respectively while both the values of fitting the second operational conditions are calculated as 0.23 and 0.18. In terms of the out-of-sample prediction, the prediction horizon is set as 150 [57,58] which is consistent with the prediction step set in the Section 3.4 for the VARX model. The performance of the FGM for out-of-sample prediction (Shown in Figure 9 and Figure 10 as an example) will be reflected in the final results of the RUL prediction.

### 4.5. Results on the RUL Prediction of Turbofan Units in the Test Set

**Figure 11:**
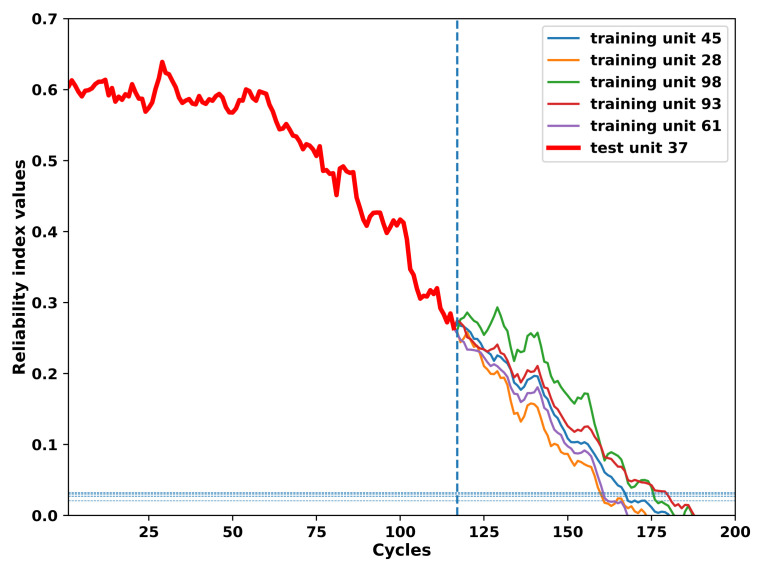
Predictions of the incomplete reliability index of the test set unit 37th considering its five most similar reliability indices of the training set units. The online prediction of the reliability index for the unit 37th in the test set is updated by future values of the external operational conditions which are predicted by means of the FGM model.

The results of the RUL prediction of all the units in the test set are obtained by means of the approach proposed in this paper. As an example, the process of the RUL prediction of the 37th unit in the test set is illustrated. According to the Section 4.2, Section 4.3, Section 4.4, the future values of operational conditions predicted by means of the FGM model are employed to update VARX predictions of the generated reliability indices of the 37th test set unit based on the trained VARX models of the units in the training set which have most similar reliability indices to the incomplete reliability index of the 37th test set unit according to results of the similarity matching. For example, according to the Figure 5, the VARX models trained based on the 45th, 28th, 98th, 93rd and 61st unit in the training set are employed to predict the future reliability index values of the 37th unit in the test set with future values of the exogenous variables namely the operational conditions updated by the FGM. The prediction process will be stopped if the future reliability index values of the test set unit 37th reach the pre-defined threshold which is shown in the horizontal dashed lines in the Figure 11. More specifically, based on the failure records of the 45th, 28th, 98th, 93rd and 61st unit in the training set, the corresponding RULs of the 37th test set unit are predicted as 54, 46, 31, 62 and 31 repsectively. Afterwards the final RUL of the 37th test unit is calculated by the average of the five RUL values obtained earlier and is determined as 44 while the truth RUL is 21 for the 37th test unit.

**Figure 12:**
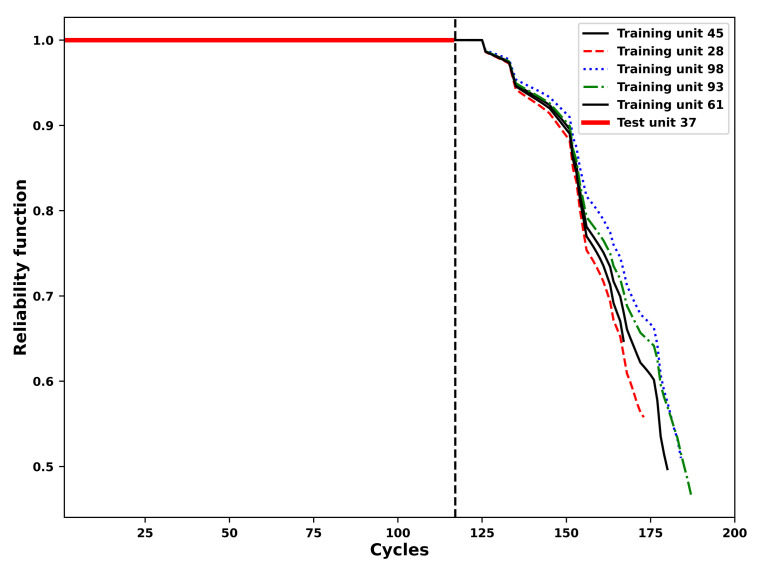
Predictions of the reliability function of the test set unit 37th considering its five most similar reliability indices of the training set units.

**Figure 13:**
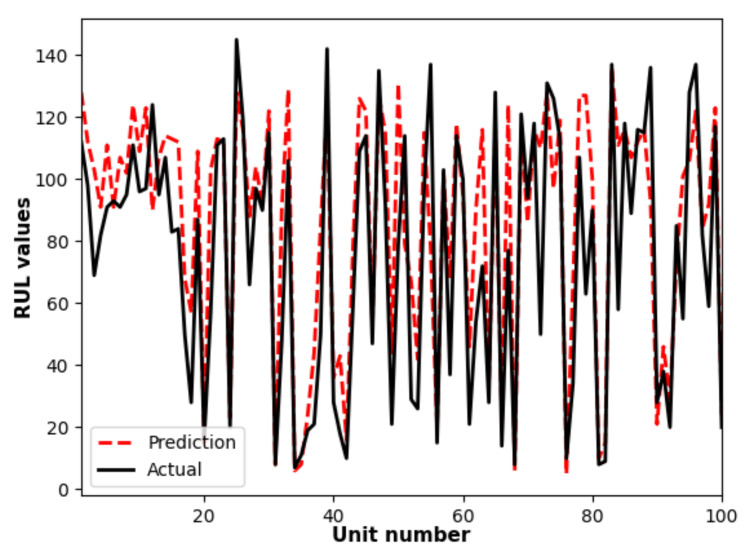
Comparision between the actual RULs and their prediction values.

According to the Section 2.2, after the reliability index is estimated the predicted reliability functions of the 37th test unit based on the 45th, 28th, 98th, 93rd and 61st training units are shown in Figure 12. Finally the comparison between actual values and the predicted values of the RULs is shown in Figure 13, which indicates the competence of the proposed approach to predict RULs for turbofan units in the test set.

## 5. Discussion

The method proposed by this paper is implemented on all the 100 test units in the FD001 data set to predict the RULs of all the test units for comparison with results which are obtained by other papers. More specifically the Root Mean Square Error (RMSE) is employed in this paper for reasonable comparisons between the prediction accuracy of the RUL by means of different methods. The results for the comparison are shown in Table 2. As shown in Table 2, the prediction accuracy of the proposed method is higher than that obtained by some benchmarking approaches, but there is still room for the prediction accuracy of the proposed approach to be improved.

The Cox PHM with time-varying covariates employed is able to generate the reliability indices and reliability functions for heterogenous turbofan units in the test set. Furthermore, the pairwise CGC tests implemented on different sensor signals of a specific turbofan unit, which also considers the influence of the external conditions, namely the operational conditions, are able to reveal mutual causal relationships between multivariate sensor signals, as shown in Figure 4. Because the sensors are arranged in different parts of a turbofan unit, the causal graphs that are shown in Figure 4 can, to a large extent, reflect the potential failure mechanism and the root cause of the failure. However, there are two main limitations to the proposed method. Considering the VARX model is a linear time series model, which requires stationarity of the processed time series, it is incapable of modelling and analysing nonlinear relationships between different sensor signals and abrupt changes in turbofan degradation behaviour. Although the VARX model can be replaced by the similarity-based method for the RUL prediction for an engineering practice in this case, the solutions to such a problem are still needed. Furthermore, in terms of the long-term predictions of the external conditions, namely the operational conditions of different turbofan test units in this paper, such predictions by means of the FGM may exhibit a high level of uncertainties that needs to be addressed in the future research.

## 6. Conclusions

To yield a real-time estimate of the reliability index and the reliability function for the RUL prediction of individual engineered systems performing their functions under time-varying external conditions, mainly the operational conditions, in this case a hybrid approach that is based on the Cox PHM with time-varying covariates and the VARX model is proposed. The proposed approach is competent for reflecting the influence of time-varying operational conditions on the degradation behaviour and, therefore, the RUL of engineered systems. In this paper, both the reliability indices of different units in the training sets under different operational conditions are generated by the Cox PHM with time-varying covariates. Afterwards, the VARX model with its variable selection that is implemented by the pairwise Conditional Granger Causality (CGC) tests is utilised to predict the reliability indices and reliability functions of units in the test set and, therefore, the corresponding RULs of units in the test set. During the RUL prediction, the Grey Model with Fourier series calibration (FGM) is employed to forecast the time series of the external conditions, namely the operational conditions of different test units, which updates the RUL prediction based on the VARX models. Finally, the RULs of all the 100 units in the FD001 test set are obtained, which shows the high accuracy of the proposed approach when forecasting the RULs. However, as mentioned earlier, there exist some limitations when applying the proposed approach to the C-MAPSS FD001 data sets for the RUL prediction, which include the linear hypothesis of the VARX model and the uncertainties of long-term predictions by means of the FGM. Such limitations will be focused on later, based on the whole C-MAPSS data set for a more accurate RUL estimate.

## Figures and Tables

**Table 1:** Fitting results of the Cox PHM with time-varying covariates based on the run-to-failure records of training set units.

Variable	Hazard Ratio	Lower 95%	Upper 95%	z	*p* Value	Partial Log-Likelihood	Penaliser
PC 1	3.59	2.67	4.83	8.43	<0.005	−328.05	0.01
PC 2	1.61	0.87	2.98	1.50	0.13		
PC 3	0.71	0.26	1.94	−0.66	0.51		

**Table 2:** Comparisons between the Root Mean Square Error (RMSE) calculated by the proposed approach and other papers.

Methods	VAR(42) [82]	SVM&ARIMA [82]	FPCA [83]	Proposed Method	CNN [84]
**RMSE**	47.63 *	39.68 *	28.06	23.35	18.45

* Corresponds to the best model proposed in the paper. FPCA and CNN correspond to the Functional Principal Component Analysis and the Convolutional Neural Network respectively.

## Data Availability

The data presented in this study are openly available in NASA Ames Prognostics Data Repository at http://ti.arc.nasa.gov/project/prognostic-data-repository.
